# Frequency and Clinical Associating Factors of Valvular Heart Disease in Asymptomatic Korean Adults

**DOI:** 10.1038/s41598-019-53277-0

**Published:** 2019-11-14

**Authors:** Min Sun Kim, Soo Jin Cho, Sung-Ji Park, Sung Won Cho, Soo-Hee Choi, Hye Seung Kim, Keumhee Carriere, Eun Kyoung Kim, Sung-A Chang, Sang-Chol Lee, Seung Woo Park

**Affiliations:** 1 Division of Cardiology, Department of Medicine, Cardiovascular Imaging Center, Heart Vascular Stroke Institute, Samsung Medical Center, Sungkyunkwan University School of Medicine, Seoul, Republic of Korea; 2 Department of Medicine, Center for Health Promotion, Samsung Medical Center, Sungkyunkwan University School of Medicine, Seoul, Republic of Korea; 3 Biostatistics Team, Statistics and Data Center, Samsung Medical Center, Seoul, Republic of Korea; 4 Department of Mathematical and Statistical Sciences, University of Alberta, Edmonton, AB Canada; 5 Present Address: Gangnam CHA Medical Center, CHA university School of Medicine, Seoul, Republic of Korea

**Keywords:** Valvular disease, Health care economics

## Abstract

Limited information is available on the prevalence and clinical determinants of valvular heart disease (VHD) in apparently healthy people. This study sought to assess the frequency and clinical associating factors of aortic stenosis (AS), aortic regurgitation (AR), mitral stenosis (MS), mitral regurgitation (MR), tricuspid regurgitation (TR) in asymptomatic individuals with health check-up examination. We included 23,254 subjects ≥50 years of age who underwent a health check-up examination with transthoracic echocardiography (TTE) between 2012 and 2016 in a single tertiary-care hospital in Korea. Among a total of 23,254 subjects, 15,358 men (66.0%) and 7,896 women (34.0%) underwent TTE. Newly identified (predominantly mild) VHD was detected in 9.4% of subjects. The most common VHD were TR (4.6%), AR (3.0%) and MR (2.4%). Clinically significant (more than moderate) VHD was identified in 176 subjects (0.8%). Age ≥75 years was associated with all clinically significant VHD, and female gender was associated with AR, MS and TR. Korea has been very active in the health check-up examination including echocardiography. We find that VHD in apparently healthy people is not uncommon than believed; all VHD except MS were more frequent in elderly over 75 years of age in a large population-based study.

## Introduction

Valvular heart disease (VHD) is expected to increase in frequency as the population ages^1^. VHD is an important cause of heart failure, arrhythmia, reduced functional capacity and early mortality. Population aging, earlier diagnosis, increased life expectancy and the availability of surgical and percutaneous interventions have led to major increases in the health care resources required for optimal management^2^. However, only limited information is available regarding the clinical and epidemiological characteristics of VHD in healthy people.

In this study, we assess the frequency and clinical associating factors of aortic stenosis (AS), aortic regurgitation (AR), mitral stenosis (MS), mitral regurgitation (MR), and tricuspid regurgitation (TR) in a large sample of apparently healthy Korean men and women undergoing health check-up examinations.

## Methods

### Study population

We retrieved data for all subjects who underwent a comprehensive health check-up examination, including transthoracic echocardiography (TTE) between 2012 and 2016 at the Health Promotion Center of Samsung Medical Center in Seoul, South Korea. In South Korea, health check-up examinations are implemented as national health insurance for all adults aged 40 or older and transthoracic echocardiography was routinely included as part of the health examination program to all who voluntarily visited our center for health examinations. Individuals who undergo health check-ups are primarily over 50 years old. Moreover, the incidence of VHD increases after the age of 50 and the prevalence of VHD increases markedly after the age of 65 years^3^. Therefore, we targeted subjects aged 50 or older in this retrospective study. We excluded subjects who had any of the following conditions at baseline: (1) history of open heart surgery including any valve surgery; (2) any self-reported cardiac symptoms such as dyspnea on exertion, chest pain, palpitation or irregular heartbeats, edema, and orthopnea; (3) limited evaluation due to subject’s poor echo window.

Since our objective was to evaluate the frequency and clinical associating factors of VHD in apparently healthy Korean adults, the analysis was restricted to subjects who underwent an echocardiography at baseline. For subjects who underwent repeated health check-up examinations during the study period, we used the data from the first examination.

The study was approved by the Institutional Review Board of Samsung Medical Center, Seoul, Korea, which waived the requirement for informed consent.

### Data collection

Subjects completed a self-administered health questionnaire that included questions on their histories of hypertension, diabetes, cardiovascular disease, and medication. Height and weight were measured and body mass index (BMI) was calculated. Hypertension was identified when subjects had a systolic blood pressure of 140 mmHg or higher, a diastolic blood pressure of 90 mmHg or higher, or were currently taking an anti-hypertension drug^4^. Diabetes was defined as serum fasting glucose level ≥126 mg/dL, hemoglobin A1c level ≥6.5%, or use of insulin or anti-diabetic medications^5^.

Blood samples were collected from the antecubital vein after overnight nil per os (NPO). Total cholesterol, triglyceride, high density lipoprotein-cholesterol (HDL-C), low density lipoprotein-cholesterol (LDL-C), fasting glucose, and hemoglobin A1c(HbA1c) levels were measured. Data was extracted from the Clinical Data Warehouse Dawin-C of Samsung Medical Center for this study.

Health-risk behaviors, including cigarette smoking and alcohol use, were also investigated in the self-reported questionnaire, but it was excluded from the analysis because of the large number of missing data and lack of quantification.

### Transthoracic echocardiography (TTE)

Standard two-dimensional TTE and Doppler echocardiography using multiple windows were conducted as part of a health promotion program using commercially available equipment (Vivid7; GE Medical Systems, Horten, Norway and SC2000; Siemens Medical Solution, Mountain view, USA). Image acquisition and measurements were performed according to the 2003 American Society of Echocardiography (ASE) guidelines and 2009 European Association of Echocardiography/ASE guidelines^6,7^. The severity of VHD was determined using an integrated approach and defined as none, mild, mild to moderate, moderate, moderate to severe, and severe (Supplementary Table S1). Clinically significant VHD was defined as moderate or more severe VHD. The primary outcome was a presence of VHD, indicated by mild or significant AS, AR, MS, MR, or TR.

The mean transaortic pressure gradient and peak transaortic velocity were measured in all views possible (that is, apical five- or three chamber and right parasternal views) and the highest values were used for analysis. The time-velocity integral at the aortic valve and the left ventricular outflow tract level were obtained by using continuous wave and pulse wave Doppler echocardiography, respectively, and the aortic valve area was calculated using the continuity equation with the parameters mentioned previously. The mean of three consecutive Doppler signals was used.

Severity of AR was determined using an integrated approach that included the size of the regurgitant jet in the left ventricular cavity, size of the vena contracta, jet deceleration rate, magnitude of the diastolic flow reversal in descending aorta, and when available, regurgitant volume (RV) and effective regurgitant orifice area (ERO).

We assessed mitral valve area by direct planimetry or, when planimetry was not feasible, the Doppler pressure half-time method. The mean diastolic transmitral pressure gradient was calculated via continuous wave Doppler echocardiography.

Severity of MR was rated based on the presence of systolic flow reversal in the pulmonary vein, size of the vena contracta, RV and ERO. One or more available measures were used for MR grading.

We conducted TR quantification by the proximal flow convergence (PISA) method. Color Doppler images of TR PISA were obtained from apical or para-apical views, zooming in on the region of interest. Size of the vena contracta and presence of systolic flow reversal in the hepatic vein were also evaluated.

### Statistical analysis

Continuous data are reported as the mean and standard deviation, and categorical data as the total sample size (n) and relative (%) frequency. For each type of VHD, Kruskal-Wallis test or Fisher’s exact test explored relationships between the 5 severity groups of the disease and quantitative or categorical variables, respectively. Then, we defined a binary level event for the severity by combining moderate to severe conditions as the primary outcome for each disease. As a preliminary investigation to find relevant variables, we performed univariable logistic regression analyses to associate VHD with demographic and clinical variables. Sparse outcomes may limit full multivariable analyses. However, perhaps because we mostly have healthy adult data, most predictors were not significantly related to the event. Finally, we analyzed the dichotomized outcome of diseases in the multivariable logistic regression to adjust for age and sex, along with a few significantly relevant predictors. All analyses were performed using SAS version 9.4 (SAS Institute, Cary, NC) and the R 3.5.1 statistical software (Vienna, Austria). P values < 0.05 were considered statistically significant but a large sample effect was considered in our discussion of the results.

## Results

Among 23,254 subjects who were included in the analysis, 15,358 males (66.0%) and 7,896 females (34.0%) underwent a health check-up examinations including TTE. Newly identified (predominantly mild) VHD was detected in 2,178 (9.4%) of subjects, and 294 (1.3%) had two or more valve diseases simultaneously. Of those 294, there were only 3 subjects with valvular stenosis (AS and MS), 198 subjects with more than two type of valvular regurgitation and 93 subjects with both valvular stenosis and regurgitation. MR together with TR, which were observed in 76 subjects, was the most common type of multi-valvular heart disease (Supplementary Table S2). TR (n = 1079, 4.6%) was the most prevalent type, followed by AR (n = 688, 3.0%) and MR (n = 551, 2.4%). Table 1 demonstrate baseline clinical characteristics of all valvular heart disease.

**Table 1:** Descriptive Clinical characteristics of valvular heart disease.

	VHD (+)	VHD(−)
	(n = 2,178)	(n = 21,076)
Male	1041 (47.8)	14317 (67.9)
Age ≥75	303 (13.9)	731 (3.47)
BMI, kg/m2	23.2 ± 3.0	24.3 ± 2.8
HTN	789 (36.9)	7182 (34.9)
DM	259 (12.1)	2782 (13.5)
Laboratory finding		
Glucose, mg/dL	98 ± 18	101 ± 20
Total cholesterol, mg/dL	189 ± 36	193 ± 36
TG, mg/dL	103 ± 55	122 ± 73
HDL-C, mg/dL	61 ± 17	57 ± 15
LDL-C, mg/dL	118 ± 32	123 ± 33
HbA1c, %	5.7 ± 0.7	5.8 ± 0.7

Numbers are presented as number (%) or mean ± standard deviation.

BMI – body mass index, HTN – hypertension, DM – diabetes mellitus, TG – triglyceride, HDL-C – high density lipoprotein-cholesterol, LDL-C – low density lipoprotein-cholesterol, HbA1c – hemoglobin A1c.

**Figure 1:**
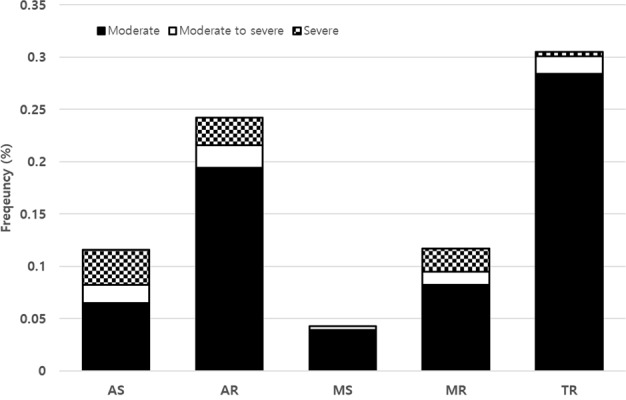
The frequency of clinically significant (more than moderate) valve abnormalities in asymptomatic Korean adults with newly diagnosed valvular heart disease.

Clinically significant (more than moderate) undiagnosed VHD was identified in 176 subjects (0.8%). There were 25 subjects (0.1%) with clinically significant valvular stenosis, 140 subjects (0.6%) with clinically significant valvular regurgitation and 11 subjects diagnosed with both clinically significant valvular stenosis and regurgitation (Supplementary Table S3). Among cases of clinically significant VHD, TR (n = 71) was the most common followed by AR (n = 56) (Fig. 1). However, among in 18 subjects who diagnosed asymptomatic severe VHD, eight were diagnosed with severe AS, 6 with severe AR, 5 with severe MR, and only one with severe TR. Two subjects had severe AS and severe AR simultaneously. On the other hand, there were no cases of severe MS. The clinical characteristics of subjects in each type of VHD are summarized in Tables 2–4. In Tables 2–4, subjects with multi-valvular heart disease were duplicated, but they were classified according to involved valve to show clinical characteristics of each valve disease. Age was positively related to the severity of VHD (p < 0.001), except in cases of MS.

**Table 2:** Clinical characteristics and severity of aortic valve disease.

	Aortic Stenosis						*P* value
	None (n = 23091)	Mild (n = 124)	Mild to moderate (n = 12)	Moderate (n = 15)	Moderate to severe (n = 4)	Severe (n = 8)	
**Female**	7849 (34.0)	35 (28.2)	3 (25.0)	4 (26.7)	2 (50.0)	3 (37.5)	0.683
**Age ≥75**	992 (4.3)	35 (28.2)	3 (25.0)	1 (6.7)	1 (25.0)	2 (25.0)	<0.001
BMI, kg/m^2^	24.1 ± 2.8	24.6 ± 3.1	24.0 ± 2.3	25.0 ± 3.0	23.6 ± 5.1	25.7 ± 7.3	0.330
**HTN**	7880 (35.0)	74(61.7)	7 (63.6)	5 (33.3)	2 (50.0)	3 (37.5)	<0.001
**DM**	3005 (13.3)	30(25.0)	2 (18.2)	1 (6.67)	1 (25.0)	2 (25.0)	0.005
Glucose, mg/dL	101 ± 20	105 ± 22	118 ± 43	97 ± 18	115 ± 21	100 ± 16	0.084
**Cholesterol, mg/dL**	192 ± 36	182 ± 38	175 ± 52	195 ± 51	168 ± 34	177 ± 44	0.01
**TG, mg/dL**	120 ± 72	107 ± 53	102 ± 58	103 ± 47	89 ± 24	79 ± 43	0.023
HDL-C, mg/dL	57 ± 15	56 ± 14	55 ± 20	58 ± 15	64 ± 6	62 ± 24	0.657
**LDL-C, mg/dL**	122 ± 33	114 ± 34	107 ± 46	126 ± 42	96 ± 38	105 ± 40	0.006
**HbA1c, %**	5.8 ± 0.7	6.1 ± 0.9	6.5 ± 1.9	5.8 ± 0.7	5.7 ± 1.0	6.0 ± 1.0	<0.001
	**Aortic Regurgitation**						***P*** **value**
	**None (n** = **22566)**	**Mild (n** = **527)**	**Mild to moderate (n** = **105)**	**Moderate (n** = **45)**	**Moderate to severe (n** = **5)**	**Severe (n** = **6)**	
**Female**	7655 (33.9)	200(38.0)	34 (32.4)	7 (15.6)	0 (0)	0 (0)	<0.001
**Age** ≥ **75**	895 (4.0)	102 (19.4)	27 (2.6)	7 (15.6)	2 (40.0)	1 (16.7)	<0.001
BMI, kg/m^2^	24.2 ± 2.9	23.9 ± 2.7	24.0 ± 3.1	24.2 ± 3.0	24.1 ± 1.8	23.3 ± 2.3	0.207
**HTN**	7325 (34.4)	573 (47.0)	47 (46.1)	21 (48.8)	2 (50.0)	3 (50.0)	<0.001
DM	2851 (13.4)	172 (14.1)	12 (11.8)	6 (14.0)	0 (0)	0 (0)	0.937
Glucose, mg/dL	101 ± 20	99 ± 17	98 ± 16	100 ± 20	106 ± 17	105 ± 17	0.465
**Cholesterol, mg/dL**	192 ± 36	187 ± 35	188 ± 36	187 ± 40	169 ± 28	198 ± 34	0.007
**TG, mg/dL**	120 ± 72	113 ± 55	108 ± 64	119 ± 62	77 ± 24	62 ± 9	0.008
HDL-C, mg/dL	57 ± 15	57 ± 16	59 ± 15	56 ± 15	56 ± 11	67 ± 27	0.743
**LDL-C, mg/dL**	123 ± 33	118 ± 32	117 ± 32	119 ± 32	103 ± 38	125 ± 42	0.010
HbA1c, %	5.8 ± 0.7	5.8 ± 0.6	5.7 ± 0.5	5.7 ± 0.6	5.7 ± 0.5	5.4 ± 0.3	0.396

Numbers are presented as number (%) or mean ± standard deviation.

BMI – body mass index, HTN – hypertension, DM – diabetes mellitus, TG – triglyceride, HDL-C – high density lipoprotein-cholesterol, LDL-C – low density lipoprotein-cholesterol, HbA1c – hemoglobin A1c

The p-value denotes statistical significance comparing the 5 groups over the condition of “none” to “severe” based on Kruskal-Wallis Test for continuous variable and Fisher’s exact test for categorical variables

The variables in bold indicate statistical significance (p < 0.05).

**Table 3:** Clinical characteristics and severity of mitral valve disease.

	Mitral Stenosis						*P* value
	None (n = 23215)	Mild (n = 26)	Mild to moderate (n = 3)	Moderate (n = 9)	Moderate to severe (n = 1)	Severe (n = 0)	
**Female**	7869 (33.9)	18 (69.2)	2 (66.7)	6 (66.7)	1 (100)		<0.001
Age ≥75	1032 (4.45)	2 (7.7)	0 (0)	0 (0)	0 (0)		0.490
**BMI, kg/m**^**2**^	24.1 ± 2.9	23.3 ± 2.4	24.6 ± 3.0	21.4 ± 2.4	21.2		0.027
**HTN**	7964 (35.1)	5 (20.8)	2 (66.7)	0 (0)	0 (0)		0.039
DM	3037 (13.4)	4 (16.7)	0 (0)	0 (0)	0 (0)		0.780
Glucose, mg/dL	100 ± 20	96 ± 15	93 ± 4	90 ± 12	101		0.194
Cholesterol, mg/dL	192 ± 36	189 ± 46	153 ± 35	197 ± 43	242		0.225
TG, mg/dL	120 ± 72	93 ± 49	78 ± 34	93 ± 26	123		0.091
HDL-C, mg/dL	57 ± 15	61 ± 15	63 ± 4	64 ± 15	59		0.202
LDL-C, mg/dL	122 ± 33	118 ± 42	79 ± 28	125 ± 39	162		0.112
HbA1c, %	5.8 ± 0.7	5.6 ± 0.6	5.6 ± 0.4	5.7 ± 0.4	5.5		0.908
	**Mitral Regurgitation**						***P*** **value**
	**None (n** = **22703)**	**Mild (n** = **469)**	**Mild to moderate (n** = **55)**	**Moderate (n** = **19)**	**Moderate to severe (n** = **3)**	**Severe (n** = **5)**	
**Female**	7595(33.5)	264 (56.3)	24 (43.6)	11 (57.9)	0 (0)	2 (40)	<0.001
**Age ≥ 75**	961 (4.23)	56 (5.42)	10 (18.2)	6 (31.6)	0 (0)	1 (20.0)	<0.001
**BMI, kg/m**^**2**^	24.2 ± 2.8	23.3 ± 2.8	23.7 ± 2.5	23.9 ± 2.9	24.3 ± 2.1	22.8 ± 4.2	<<0.001
HTN	7360 (35.0)	576 (36.2)	22 (40.7)	8 (42.1)	2 (66.7)	3 (60.0)	0.401
**DM**	2856 (13.6)	174 (10.9)	8 (14.8)	2 (10.5)	1 (33.3)	0 (0)	0.033
**Glucose, mg/dL**	100 ± 20	97 ± 18	98 ± 15	93 ± 15	98 ± 12	92 ± 8	<0.001
**Cholesterol, mg/dL**	192 ± 36	189 ± 36	185 ± 35	169 ± 36	181 ± 22	202 ± 29	0.047
**TG, mg/dL**	121 ± 72	101 ± 53	97 ± 47	90 ± 34	88 ± 6	108 ± 52	<0.001
**HDL-C, mg/dL**	57 ± 15	61 ± 17	60 ± 18	56 ± 19	65 ± 15	57 ± 8	<0.001
**LDL-C, mg/dL**	122 ± 33	118 ± 32	115 ± 32	104 ± 28	106 ± 13	135 ± 27	0.002
HbA1c, %	5.8 ± 0.7	5.7 ± 0.6	5.7 ± 0.6	5.6 ± 0.6	6.0 ± 0.6	5.6 ± 0.1	0.744

Numbers are presented as number (%) or mean ± standard deviation.

BMI – body mass index, HTN – hypertension, DM – diabetes mellitus, TG – triglyceride, HDL-C – high density lipoprotein-cholesterol, LDL-C – low density lipoprotein-cholesterol, HbA1c – hemoglobin A1c

Please see the legend for Table 2.

**Table 4:** Clinical characteristics and severity of tricuspid regurgitation.

	Tricuspid Regurgitation						*P* value
	None (n = 22175)	Mild (n = 879)	Mild to moderate (n = 129)	Moderate (n = 66)	Moderate to severe (n = 4)	Severe (n = 1)	
**Female**	7196 (32.5)	567 (64.5)	85 (65.9)	46 (69.7)	2 (50)	0 (0)	<0.001
**Age ≥75**	897 (4.1)	96 (10.9)	23 (17.8)	13 (19.7)	4 (100)	1 (100)	<0.001
**BMI, kg/m**^**2**^	24.3 ± 2.8	22.8 ± 2.8	21.7 ± 2.8	22.0 ± 3.2	22.2 ± 2.0	24	<0.001
**HTN**	7235 (35.7)	676 (30.5)	33 (26.2)	25 (37.9)	1 (33.3)	1 (100)	<0.001
**DM**	2809(13.9)	211(9.5)	14 (11.1)	5 (7.6)	1 (33.3)	1 (100)	<0.001
**Glucose, mg/dL**	101 ± 20	96 ± 16	97 ± 26	95 ± 15	93 ± 17	78	<0.001
Cholesterol, mg/dL	192 ± 36	191 ± 35	189 ± 35	194 ± 37	150 ± 33	168	0.270
**TG, mg/dL**	121 ± 72	98 ± 54	88 ± 47	88 ± 46	91 ± 29	55	<0.001
**HDL-C, mg/dL**	57 ± 15	63 ± 17	64 ± 20	63 ± 20	52 ± 12	88	<0.001
**LDL-C, mg/dL**	122 ± 33	119 ± 31	118 ± 31	122 ± 30	86 ± 37	84	0.013
HbA1c, %	5.8 ± 0.7	5.7 ± 0.5	5.8 ± 0.8	5.7 ± 0.7	5.7 ± 0.4	7	0.289

Numbers are presented as number (%) or mean ± standard deviation.

BMI – body mass index, HTN – hypertension, DM – diabetes mellitus, HbA1c – hemoglobin A1c, TG – triglyceride, HDL-C – high density lipoprotein-cholesterol, LDL-C – low density lipoprotein-cholesterol.

Please see the legend for Table 2.

**Figure 2:**
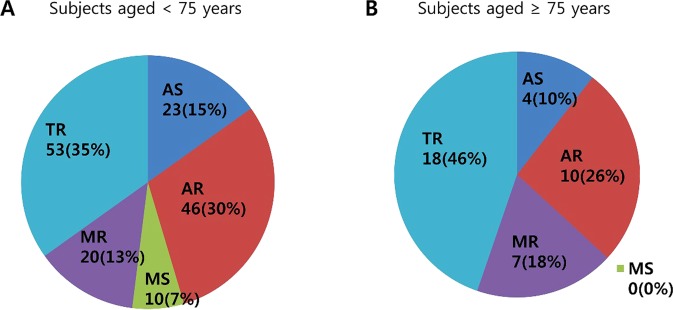
The distribution of clinically significant valvular heart disease according to the age. (**A**) The distribution of clinically significant (more than moderate) valvular heart disease in subjects aged <75 years. (**B**) The distribution of clinically significant (more than moderate) valvular heart disease in subjects aged ≥75 years.

Univariable logistic analyses were performed for clinically significant VHD (more than moderate) (Supplementary Table S4). Previous prospective studies of subjects aged 65 years and older have demonstrated that the proportion of patients with clinically significant VHD was increased in subjects aged 75 years or older^8^. To explore the effects on aging, we dichotomized the subjects based on their age less than or at least 75 years old. Age ≥75 years was associated with all clinically significant VHD, and female gender was associated with AR, MS and TR. The results were also consistent in the multivariable analysis. Details on the associating factors are provided in Table 5 and Supplementary Table S4. When the frequency of clinically significant VHD was examined in subjects by age groups by 75 years, TR was the most frequent form of VHD in both groups (Fig. 2). There were 1034 subjects who were older than 75 years old. While the frequency of newly diagnosed VHD in all study population was 9.4%, the frequency was higher in those aged ≥75 years (29.3%) than those aged <75 years, which was 8.4%. A total of 34 subjects (3.3%) were diagnosed clinically significant VHD in subjects who were older than 75 years old. Among them, two clinically significant VHD were simultaneously present in 5 subjects; AS with AR, AS with TR, AR with MR, AR with TR, and MR with TR. None of the subjects were diagnosed with more than 3 types of clinically significant VHD.

**Table 5:** Clinical associating factors by types of more than moderate valvular heart diseases based on multivariable logistic regressions.

	Odds ratio	95% CI	P value
**Aortic stenosis**			
Sex (Female)	0.98	0.45–2.13	0.962
Age (≥75years)	4.13	1.52–11.26	0.006
**Aortic regurgitation**			
Sex (Female)	0.29	0.13–0.62	0.001
Age (≥75years)	5.08	2.60–9.94	<0.0001
**Mitral stenosis**			
Sex (Female)	4.20	1.21–14.56	0.024
Age (≥75years)	0.96	0.06–15.54	0.978
**Mitral regurgitation**			
Sex (Female)	1.75	0.84–3.65	0.137
Age (≥75years)	7.73	3.36–17.77	<0.0001
**Tricuspid regurgitation**			
Sex (Female)	3.57	2.17–5.87	<0.0001
Age (≥75years)	7.08	4.11–12.19	<0.001
BMI (≥25 kg/m^2^)	0.51	0.28–0.92	0.027

Age and sex were retained in all models, and other covariates considered before eliminating for statistical insignificance include: hypertension, diabetes mellitus, BMI, glucose, cholesterol, triglyceride, high density lipoprotein-cholesterol, low density lipoprotein-cholesterol, and hemoglobin A1c (See Supplementary Table S4). CI – confidential interval, BMI – body mass index.

## Discussion

In this study, based on a large study population of asymptomatic Korean adults aged >50 years, we found that the frequency of VHD was 9.4%, which was higher than that observed in Western studies. Previous studies in the West have varied widely in their estimates of the prevalence of VHD, as the overall prevalence of VHD in the United States is 2.5% with wide age-related variation^1,9^. On the other hand, some studies detected previously undetected VHD in just over half of 2500 participants aged >65 years^8^. Consistent with these results, we observed a significantly higher rate of VHD in subjects aged ≥75 years. While the frequency of newly identified VHD in subjects aged <75 years was 8.4%, the frequency was higher in those aged ≥75 years (29.3%). The use of a study population over 50 years of age is expected to result in a higher apparent frequency of VHD compared to previous studies.

The frequency and severity of all types of VHD except MS were higher in the elderly. Our findings that age significantly affects the prevalence of VHD are consistent with those of previous studies^8,10,11^. A previous study based on data derived from the Korean National Health Insurance Service between 2006 and 2011, Jang *et al*. reported that the overall age-standardized cumulative prevalence of non-rheumatic valve disease was increased in patients over 65 years of age^12^. Although the present study could not provide information regarding the prevalence of VHD because we obtained health screening data from a single center, our results demonstrate the possibility of identifying asymptomatic VHD and its associating factors in apparently healthy Korean adults, with applications to real world practice.

Regarding the distribution of clinically significant VHD (Fig. 2), the frequency of AS was relatively lower in subjects ≥75 years than in those <75 years. However, based on the distribution of all VHD, AS and AR were more frequent in subjects ≥75 years (Supplementary Fig. S2). There are several possible explanations for this point. Degenerative VHD represents the most common etiology of AS in the elderly population. Most of them have only mild thickening and normal valve function, called aortic sclerosis, or mild AS. Moreover, this study is based on asymptomatic Korean adults aged >50 years. Thus, it is likely that there are relatively small number of patients with clinically significant AS.

Although the association between hypertension and AR was significant (OR 1.78(1.04–3.06)), there was a strong association between AR and age (OR 4.71(2.37–9.36)) (Supplementary Table S4). Earlier reports suggested that hypertension predisposes subjects to aortic root enlargement and AR. However, other previous studies demonstrated a strong association between age and AR, which may explain the lack of association between AR and hypertension after adjusting for age in the multivariable model^13,14^. In addition, obesity was associated with a lower frequency of TR. Lower BMI has been associated with valvular regurgitation, perhaps due the increased ease of echocardiographic imaging in thin individuals^9,15^.

The frequency of clinically significant (more than moderate) VHD was also considerably higher than expected in the present study. Severe MS was not found in anyone, but a total of 18 subjects were diagnosed with asymptomatic severe VHD. Although further explorations of the contemporary prevalence and natural course of VHD is required, VHD often has a long latent period and the optimum timing of intervention requires initial identification by screening programs.

This study has several limitations. First, it was a retrospective study performed in a sample of subjects who voluntarily visited our center for health examinations and our study might reflect some selection bias, because individuals in the study population were self-referred for health examinations. It is not yet known what the difference may exist among healthy subjects who did not bother with health examinations, as the national program is not mandatory. Although we do not intend to generalize to general populations, we believe that any differences will be small and our data and results are uniquely based on health promotion initiative, including echocardiography. Second, our study subjects were all ethnically Korean, and our findings may not apply to other populations or different ethnicities. Although this study was conducted at a single center among asymptomatic subjects, we believe our large sample accurately represent healthy, asymptomatic, and older Korean adults with regard to the prevalence and severity of VHD.

## Conclusion

Korea has actively promoted health check-up examinations, including echocardiography. We found that VHD in apparently healthy people is not less uncommon than hypothesized. These data demonstrate that all forms of VHD except MS were more frequent in elderly subjects over 75 years of age in a large population-based study. Valve diseases thus represent important public-health problems.

## Supplementary information

Supplementary Table and Figure

## Data Availability

The datasets generated during and/or analysed during the current study are available from the corresponding author on reasonable request.

## References

[1] Nkomo VT (2006). Burden of valvular heart diseases: a population-based study. Lancet.

[2] d’Arcy JL, Prendergast BD, Chambers JB, Ray SG, Bridgewater B (2011). Valvular heart disease: the next cardiac epidemic. Heart.

[3] Iung B, Vahanian A (2014). Epidemiology of acquired valvular heart disease. Can J Cardiol.

[4] National High Blood Pressure Education, P. in *The Seventh Report of the Joint National Committee on Prevention, Detection, Evaluation, and Treatment of High Blood Pressure* (National Heart, Lung, and Blood Institute (US), 2004). 20821851

[5] Inzucchi SE (2012). Management of hyperglycemia in type 2 diabetes: a patient-centered approach: position statement of the American Diabetes Association (ADA) and the European Association for the Study of Diabetes (EASD). Diabetes care.

[6] Zoghbi WA (2003). Recommendations for evaluation of the severity of native valvular regurgitation with two-dimensional and Doppler echocardiography. Journal of the American Society of Echocardiography: official publication of the American Society of Echocardiography.

[7] Baumgartner Helmut, Hung Judy, Bermejo Javier, Chambers John B., Evangelista Arturo, Griffin Brian P., Iung Bernard, Otto Catherine M., Pellikka Patricia A., Quiñones Miguel (2009). Echocardiographic Assessment of Valve Stenosis: EAE/ASE Recommendations for Clinical Practice. Journal of the American Society of Echocardiography.

[8] d’Arcy JL (2016). Large-scale community echocardiographic screening reveals a major burden of undiagnosed valvular heart disease in older people: the OxVALVE Population Cohort Study. Eur Heart J.

[9] Singh JP (1999). Prevalence and clinical determinants of mitral, tricuspid, and aortic regurgitation (the Framingham Heart Study). Am J Cardiol.

[10] Biava G (1997). Prevalence of valvular regurgitation in structurally normal hearts: a colour-Doppler study. Coron Artery Dis.

[11] Akasaka T (1987). Age-related valvular regurgitation: a study by pulsed Doppler echocardiography. Circulation.

[12] Jang SY (2014). Changes in the etiology of valvular heart disease in the rapidly aging Korean population. Int J Cardiol.

[13] Kim M (1996). Effect of hypertension on aortic root size and prevalence of aortic regurgitation. Hypertension.

[14] Lonati L (1992). Prevalence of physiological valvular regurgitation in hypertensive patients: echocardiographic and color Doppler study. Cardiology.

[15] de Divitiis O (1981). Obesity and cardiac function. Circulation.

