# Publisher Correction: Frequency and Clinical Associating Factors of Valvular Heart Disease in Asymptomatic Korean Adults

**DOI:** 10.1038/s41598-020-58889-5

**Published:** 2020-02-19

**Authors:** Min Sun Kim, Soo Jin Cho, Sung-Ji Park, Sung Won Cho, Soo-Hee Choi, Hye Seung Kim, Keumhee Carriere, Eun Kyoung Kim, Sung-A. Chang, Sang-Chol Lee, Seung Woo Park

**Affiliations:** 1 Division of Cardiology, Department of Medicine, Cardiovascular Imaging Center, Heart Vascular Stroke Institute, Samsung Medical Center, Sungkyunkwan University School of Medicine, Seoul, Republic of Korea; 2 Department of Medicine, Center for Health Promotion, Samsung Medical Center, Sungkyunkwan University School of Medicine, Seoul, Republic of Korea; 3 Biostatistics Team, Statistics and Data Center, Samsung Medical Center, Seoul, Republic of Korea; 4 Department of Mathematical and Statistical Sciences, University of Alberta, Edmonton, AB Canada; 5 Present Address: Gangnam CHA Medical Center, CHA University School of Medicine, Seoul, Republic of Korea

Correction to: *Scientific Reports* 10.1038/s41598-019-53277-0, published online 14 November 2019

In the original version of this Article, Seung Woo Park was incorrectly listed as the corresponding author. The correct corresponding author for this Article is Sung-Ji Park. Correspondence and requests for materials should be addressed to tyche.park@gmail.com. This error has now been corrected in the HTML and PDF versions of the Article.

