# Supplementary material for: Frequency and Clinical Associating Factors of Valvular Heart Disease in Asymptomatic Korean Adults
Source: Sci Rep. 2019 Nov 14;9:16741. doi: 10.1038/s41598-019-53277-0 (PMC6856181; doi:10.1038/s41598-019-53277-0)
Supplement: Supplementary file 1 — Supplementary Table and Figure [file 41598_2019_53277_MOESM1_ESM.pdf]

Original article

Frequency and Clinical Associating Factors of Valvular Heart Disease in Asymptomatic Korean Adults

Min Sun Kim[1*#], Soo Jin Cho[2*], Sung-Ji Park[1], Sung Won Cho[2], Soo-Hee Choi[2], Hye Seung Kim[3], Keumhee Carriere[3,4], Eun Kyoung Kim[1], Sung-A Chang[1], Sang-Chol Lee[1], Seung Woo Park[1]

[1]Division of Cardiology, Department of Medicine, Cardiovascular Imaging Center, Heart Vascular Stroke Institute, Samsung Medical Center, Sungkyunkwan University School of Medicine, Seoul, Republic of Korea

[2]Department of Medicine, Center for Health Promotion, Samsung Medical Center, Sungkyunkwan University School of Medicine, Seoul, Republic of Korea

[3]Biostatistics Team, Statistics and Data Center, Samsung Medical Center, Seoul, Republic of Korea

[4]Department of Mathematical and Statistical Sciences, University of Alberta, Edmonton, AB, Canada

*Drs. Kim and Cho contributed equally to this work

[#]Dr. Kim is currently working in Gangnam CHA Medical Center, CHA university School of Medicine, Seoul, Republic of Korea

Corresponding Author

Sung-Ji Park, M.D., Ph.D.

Director of Cardiovascular Imaging Center

Division of Cardiology, Department of Medicine, Cardiovascular Imaging Center, Heart Vascular Stroke Institute, Samsung Medical Center, Sungkyunkwan University School of Medicine, 81 Irwon-ro, Gangnam-gu, Seoul, 06351, Korea

Table S1

Echocardiographic parameters in grading severity of VHD.

**Aortic stenosis**	**Mild**	**Moderate**	**Severe**
Aortic jet velocity (m/s)	2.6-2.9	3.0-4.0	>4.0
Mean gradient (mmHg)	<20	20-40	>40
Aortic valvular area (AVA) (cm^2)	>1.5	1.0-1.5	<1.0
Indexed AVA (cm^2/cm^2)	>0.85	0.60-0.85	<0.6
Velocity ratio	>0.50	0.25-0.50	<0.25
**Aortic regurgitation**	**Mild**	**Moderate**	**Severe**
Vena contracta width (cm)	<0.3	0.3-0.6	>0.6
Pressure half time (msec)	>500	500-200	<200
Regurgitation volume (ml/beat)	<30	30-60	≥60
Regurgitant fraction (%)	<30	30-50	≥50
EROA (cm^2)	<0.10	0.10-0.29	≥0.30
**Mitral stenosis**	**Mild**	**Moderate**	**Severe**
Valve area (cm^2)	>1.5	1.0-1.5	<1.0
Mean gradient (mmHg)	<5	5-10	>10
Pulmonary artery pressure (mmHg)	<30	30-50	>50
**Mitral regurgitation**	**Mild**	**Moderate**	**Severe**
Vena contracta width (cm)	<0.3	0.3-0.69	≥0.7
Regurgitation volume (ml/beat)	<30	30-59	≥60
Regurgitant fraction (%)	<30	30-49	≥59
EROA (cm^2)	<0.20	0.20-0.39	≥0.40
**Tricuspid regurgitation**	**Mild**	**Moderate**	**Severe**
Vena contracta width (cm)	Not defined	Not defined	>0.7
Jet area – central jets (cm^2)	<5	5-10	>10
PISA radius (cm)	<0.5	0.6-0.9	>0.9
Hepatic vein flow	Systolic dominance	Systolic blunting	Systolic reversal

EROA – effective regurgitant orifice area, PISA – proximal isovelocity surface area

Table S2

Type of all valvular heart disease.

	**VHD (n = 2178)**
TR only	898
AR only	496
MR only	391
AS only	81
MS only	18
MR+TR	76
AR+TR	65
AS+AR	49
AR+MR	39
MS+MR	6
AS+MR	5
AS+TR	5
MS+TR	3
AS+MS	3
AR+MS	1
AR+MR+TR	18
AR+MS+MR	2
AR+MS+TR	1
AS+AR+MR	5
AS+MS+AR	2
AS+AR+TR	4
AS+MR+TR	3
MS+MR+TR	1
AS+AR+MR+TR	4

Table S3

Type of clinically significant valvular heart disease.

	**Clinically significant VHD (n = 176)**
TR only	67
AR only	46
MR only	24
AS only	18
MS only	6
MR+TR	1
AR+TR	1
AS+AR	7
AR+MR	1
MS+MR	1
AS+TR	1
MS+TR	1
AS+MS	1
AR+MS	1

Table S4

Univariable analysis for more than moderate valvular heart diseases.

	**AS**<br>OR (95% CI)	**AR**<br>OR (95% CI)	**MS**<br>OR (95% CI)	**MR**<br>OR (95% CI)	**TR**<br>OR (95% CI)
Sex (Female)	0.97(0.44-2.17)	0.28 (0.13-0.61)*	4.55 (1.18-16.7)*	1.79 (0.85-3.85)	4.00 (2.44-6.67)*
Age (≥75years)	3.75(1.29-10.86)*	4.71 (2.37-9.36)*	1.02 (0.06-17.48)	7.56 (3.19-17.93)*	7.41 (4.32-12.69)*
BMI (≥25kg/m^2)	1.48(0.68-3.20)	1.13 (0.65-1.97)	0.09 (0.01-1.56)	0.61 (0.24-1.54)	0.40 (0.22-0.73)*
HTN	1.09(0.50-2.38)	1.78 (1.04-3.06)*	0.10 (0.01-1.67)	1.72 (0.81-3.65)	1.13 (0.70-1.83)
DM	1.12(0.39-3.25)	0.83 (0.35-1.93)	0.34 (0.02-5.85)	0.81 (0.24-2.68)	0.71 (0.32-1.54)
Glucose, mg/dL	1.00(0.98-1.02)	1.06 (0.58-1.91)	0.95(0.89-1.01)	0.97(0.93-1.00)	0.98(0.96-1.00)
Cholesterol, mg/dL	1.00(0.98-1.01)	1.00(0.99-1.00)	1.00(0.99-1.03)	0.99(0.98-1.00)	1.00(1.00-1.01)
TG, mg/dL	0.99(0.98-1.00)	1.00(0.99-1.00)	0.99(0.98-1.01)	0.99(0.98-1.00)	0.99(0.98-0.99)
HDL-C, mg/dL	1.01(0.99-1.04)	1.00(0.98-1.02)	1.02(0.99-1.06)	1.00(0.98-1.03)	1.02(1.01-1.04)
LDL-C, mg/dL	0.99(0.98-1.01)	1.00(0.99-1.00)	1.01(0.99-1.03)	0.99(0.98-1.00)	1.00(0.99-1.00)
HbA1c (>6.5%)	1.93(0.73-5.10)	0.88 (0.35-2.22)	0.45 (0.03-7.67)	1.27(0.38-4.28)	0.81(0.35-1.87)

*P value < 0.05 based on univariable logistic regression (Chi-square test).

AS – aortic stenosis, AR – aortic regurgitation, MS – mitral stenosis, MR – mitral regurgitation, TR – tricuspid regurgitation, OR – odds ratio, CI – confidential interval, BMI – body mass index, HTN – hypertension, DM – diabetes mellitus, TG – triglyceride, HDL-C – high density lipoprotein-cholesterol, LDL-C – low density lipoprotein-cholesterol, HbA1c – hemoglobin A1c

Supplementary Fig. S2. The distribution of all valvular heart disease according to the age.

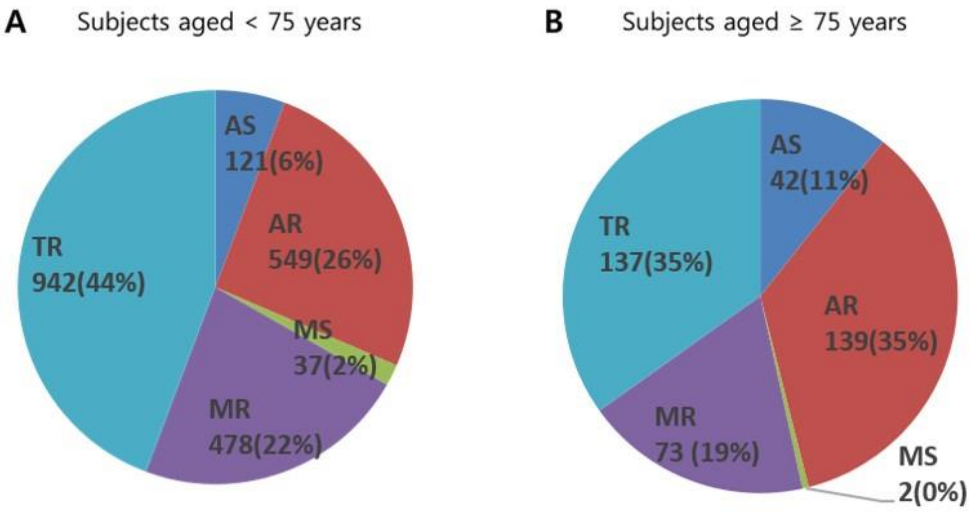

(A) The distribution of all valvular heart disease in subjects aged < 75 years. (B) The distribution of all valvular heart disease in subjects aged ≥75 years.
